# Enhancement of Bioreactor Performance Using Acclimatised Seed Sludge in Anaerobic Treatment of Chicken Slaughterhouse Wastewater: Laboratory Achievement, Energy Recovery, and Its Commercial-Scale Potential

**DOI:** 10.3390/ani11113313

**Published:** 2021-11-19

**Authors:** Tuan Nurfarhana Tuan Mohd Marzuki, Syazwani Idrus, Mohammed Ali Musa, Abdul Malek Abdul Wahab, Nur Syakina Jamali, Hasfalina Che Man, Sabrina Ng Muhamad Ng

**Affiliations:** 1 Department of Civil Engineering, Faculty of Engineering, Universiti Putra Malaysia, Serdang 43400, Malaysia; t_nurfarhana@yahoo.com (T.N.T.M.M.); alisulezee@gmail.com (M.A.M.); sabng@icloud.com (S.N.M.N.); 2 Department of Civil and Water Resources Engineering, University of Maiduguri, Maiduguri 600104, Nigeria; 3 School of Mechanical Engineering, College of Engineering, Universiti Teknologi MARA, Shah Alam 40450, Malaysia; abdmalek@uitm.edu.my; 4 Department of Chemical and Environmental Engineering, Faculty of Engineering, Universiti Putra Malaysia, Serdang 43400, Malaysia; syakina@upm.edu.my; 5 Department of Biological and Agricultural Engineering, Faculty of Engineering, Universiti Putra Malaysia, Serdang 43400, Malaysia; hasfalina@upm.edu.my

**Keywords:** anaerobic digestion, specific methane production, organic loading rate, energy yield

## Abstract

**Simple Summary:**

Chicken slaughterhouses generate wastewater rich in organic contaminants and nutrients. Chicken slaughterhouse wastewater (CSWW) is considered high-strength wastewater, hence becoming a potential candidate for treatment processes that can recover energy. This study focused on the performance comparison of two sets of upflow anaerobic sludge blanket (UASB) reactors inoculated with different seed sludge treating CSWW. The reactor inoculated with seed sludge acclimatised on modified synthetic wastewater successfully produced a significant methane yield and removed chemical oxygen demand (COD) in the effluent, which complied with the discharge parameter set by the Department of Environment (DOE) Malaysia. At the optimum loading rate of bioreactor operation, energy recovery from laboratory scale (LS) and commercial scale (CS) systems were evaluated and proven to produce significant energy yield for wet market usage. Furthermore, a preliminary design of an on-site CS system was also proposed based on the actual daily volume of CSWW generated, output from process performance study, and existing design of the LS system.

**Abstract:**

Lack of good management practice of chicken slaughterhouse wastewater (CSWW) has caused pollution into water bodies. In this study, the potential of seed sludge acclimatised modified synthetic wastewater (MSWW) on bioreactor performance and energy recovery of CSWW treatment was investigated. Two sets of upflow anaerobic sludge blanket (UASB) reactors were employed. The seed sludge in UASB 2 was acclimatised with MSWW for 30 days. In UASB 1, no acclimatisation process was undertaken on seed sludge for control purposes. After the acclimatisation process of UASB 2, both reactors were supplied with CSWW under the same condition of organic loading rate (OLR = 0.5 to 6 gCOD/L/d) and mesophilic condition (37 °C). COD removal efficiencies of UASB 2 were >80% all through the steady-state of the OLR applied. Meanwhile, a drastic decrease in overall performance was observed in UASB 1 when the OLR was increased to 3, 4, 5, and 6 gCOD/L/d. Energy recovery from laboratory scale and projected value from commercial-scale bioreactor were 0.056 kWh and 790.49 kWh per day, respectively. Preliminary design of an on-site commercial-scale anaerobic reactor was proposed at a capacity of 60 m^3^.

## 1. Introduction

The potential of renewable raw materials in energy generation through biogas formation is at the forefront of energy security discussions of recent times. Chicken slaughterhouse wastewater (CSWW) showed a high potential for energy production due to the presence of protein, lipids, fats, oil, and grease [1]. Nevertheless, industrial activities are subjected to laws and regulations that are aimed at improving effluent quality required to be released into receiving water bodies and are now more stringent than ever before. This could be due to the pollution effect of discharging untreated/improperly treated wastewater [2]. Treatment technologies such as conventional activated sludge, dissolved air floatation, lagoon and pond systems, coagulation and flocculation processes, and anaerobic digestion (AD) are frequently used for domestic wastewater treatment [3,4]. These technologies are also applied in the treatment of CSWW. Nevertheless, the major drawbacks of these technologies are the large space requirement, substantial sludge production, odour, and expensive installation. Biological processes are widely known to provide significant benefits in the treatment of wastewater in this context. AD is a process that converts wastes into biogas and enables for efficient treatment of effluents with high organic content. Biogas can be used as an alternative energy source because it is mainly made up of methane (CH_4_) and carbon dioxide (CO_2_) [5]. Moreover, the release of methane (CH_4_), carbon dioxide (CO_2_), and nitrogen oxide (N_2_O) from open lagoons and ponds contributes immensely to the deterioration of the environment [6]. Among the various treatment technologies of the wastewater, AD using upflow anaerobic sludge blanket (UASB) reactor appeared promising [7]. The advantages range from the high efficiency, flexibility, and smaller footprint, to less maintenance and low energy demand [8]. Nonetheless, the system usually requires a long hydraulic retention time (HRT) to start, due to a range of factors, including acclimatisation period of the seed sludge, variation of substrates composition, which includes inhibitory compound, upflow velocity (V_up_), and the liquid mixing. Moreover, pH and temperature are also important factors that significantly affect the performance of UASB reactors. Although anaerobic reactors can operate at three different temperature ranges (psychrophilic, mesophilic, hyperthermophilic, and thermophilic), reactors operating at low temperatures (psychrophilic) usually experience low maximum specific growth rate and methanogenic activity [3,9,10]. However, several studies suggest that maintaining the temperature at mesophilic (35 °C) condition significantly improves the performance UASB reactor, compared with the psychrophilic and thermophilic range [11]. Another important parameter is V_up_, which is directly related to HRT and entrapment of suspended solids (SSs). According to Daud et al. [12], low V_up_ entails an increase in HRT, which boosts the SS removal efficiency of the system. On the other hand, chemical oxygen demand (COD) and SS removal efficiencies usually decrease at elevated V_up_ because higher V_up_ reduces the contact time between sludge and wastewater, in addition to smashing of sludge granules, and resultantly higher washout of solids. Therefore, to achieve higher COD removal and biogas production in UASB reactor, the system must take into consideration the reactor design, seed sludge used, and operating conditions.

Most of the previous studies have highlighted bioreactor design [13,14,15,16] and organic loading rate (OLR) for CSWW treatment [17,18,19]. There are two previous studies on anaerobic treatment of CSWW that have addressed sources or types of seed sludge. Debik and Coskun [20] reported on a comparative study on two sets of bioreactors—the first bioreactor was seeded with fully anaerobic granule, and the second bioreactor contained a mixture of anaerobic non-granular biomass and granular biomass. Another study reported [21] on the effect of different sources of seed sludge in batch mode. The seed sludge was collected from a brewery wastewater treatment plant and from an anaerobic lagoon. However, no previous study in the literature has investigated the comparative performance of UASB reactors containing seed sludge acclimatised MSWW and sludge collected from domestic wastewater treatment plants. Therefore, in this study, a MSWW was used for acclimatisation of seed sludge to investigate the effect on bioreactor performance and energy recovery. This newly proposed approach was employed to reduce the long start-up period in operation of the UASB reactor [22] and to improve the development of the microbial population. Another set of UASB (UASB 1, seeded with sludge collected from domestic wastewater treatment plant) was utilised as a control for the experimental UASB (UASB 2, seeded with acclimatised sludge) reactor. Furthermore, in this work, energy recovery from laboratory scale (LS) bioreactor and projected energy recovery from commercial scale (CS) bioreactor were calculated, while most of the previous work emphasised energy recovery during manure treatment [23,24,25,26]. Therefore, there are two objectives of the present study to enhance the understanding of anaerobic treatment on CSWW as follows: (1) to investigate the effect of acclimatised seed sludge using MSWW on bioreactor performance in terms of OLR and (2) to calculate the energy recovery from LS bioreactor and projected energy recovery from CS bioreactor and propose a preliminary design of on-site CS bioreactor system based on actual daily volume of CSWW generated, output from process performance study, and existing design of LS system.

## 2. Materials and Methods

### 2.1. Physicochemical Characterisation of CSWW

CSWW was collected from a wet market located in Seri Kembangan, Selangor, Malaysia. It was then brought to the Public Health Laboratory, Civil Engineering Department, Faculty of Engineering, Universiti Putra Malaysia. At the laboratory, the collected sample was sieved to exclude any suspended particles and feathers. The sample wastewater was then analysed for COD, total suspended solids (TSSs), volatile-suspended solids (VSSs), fats, oil, and grease (FOG), pH, colour, and turbidity. Table 1 shows the composition of the CSWW wastewater collected.

### 2.2. Reactor Design

**Figure 1:**
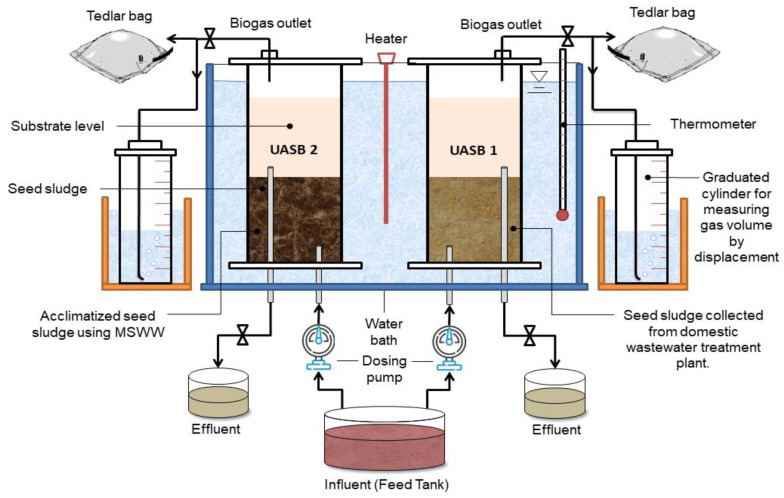
Schematic diagram of UASB 1 and 2 reactors.

**Figure 2:**
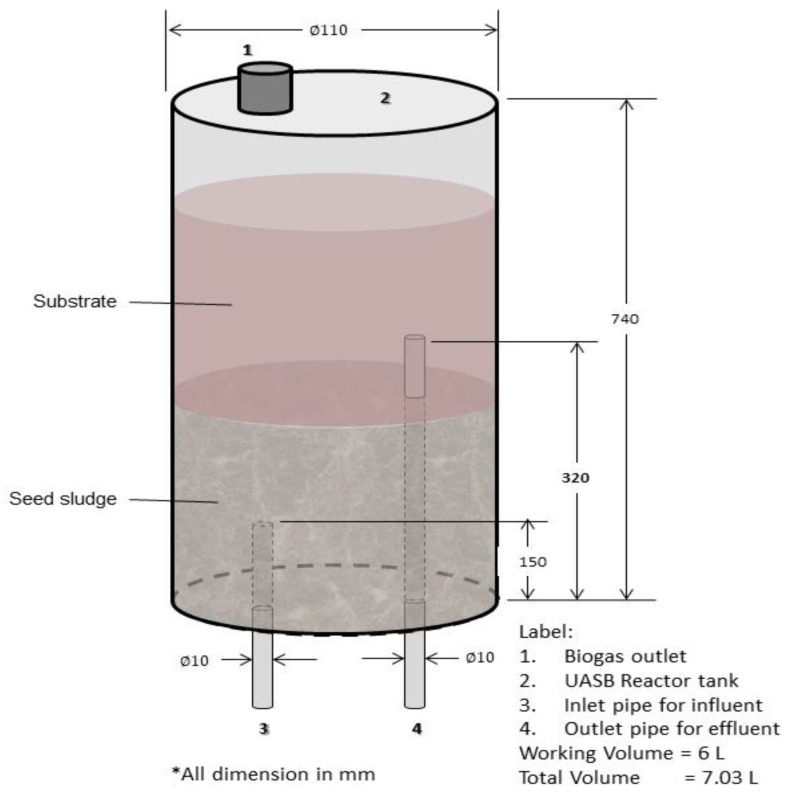
Cross section of UASB reactor.

Two UASB (1 and 2) reactors, as shown in Figure 1, were used for the investigation. Polyvinyl chloride (PVC) cylindrical tubes with an internal diameter of 110 mm and a height of 740 mm were used to construct the reactors (Figure 2). Each of the two reactors had a gas sample Tedlar bag with a capacity of 10 L linked to the gas outflow tube. Each reactor had an equally spaced effluent discharge outlet, apart from the influent inlet at the bottom and gas exit flows at the top. A thermometer was also installed in the water bath to monitor the temperature of the water bath as the heating coil was placed in between the two reactors with an immersed edge in the water bath. The reactors were run in upflow mode.

The seed sludge for UASB 2 (before acclimatisation with MSWW) and UASB 1 were collected from a domestic wastewater treatment plant, Universiti Putra Malaysia, Selangor, Malaysia, with an initial VSS concentration of 17,000 mg/L for both reactors. After acclimatisation of seed sludge on MSWW, VSS concentration in UASB 2 was 43,800 mg/L.

Behera and Ghangrekar [27] have suggested a sludge loading rate (SLR) of less than 0.3 kgCOD/kg of VSS/d for a proper start-up of a UASB reactor; hence, SLR of 0.02 kgCOD/kg VSS/d was chosen for the start-up of UASB 2 fed on MSWW. After 30 days of acclimatisation, SLR for UASB 2 fed on CSWW was 0.021 kgCOD/kg VSS/d and UASB 1 (without acclimatisation), the SLR applied was 0.051 kgCOD/kg VSS/d, where both SLR were less than 0.3 kgCOD/kg VSS/d, as suggested earlier. SLR for UASB 2 was slightly lower because UASB 1 and UASB 2 were fed with CSWW concurrently with the same OLR, and the VSS concentration of seed sludge in UASB 2 was higher after acclimatisation, as reported earlier. The OLR was increased gradually in each phase for both reactors after 14–18 days. The reactor was fed from the bottom through a sludge blanket using a dosing pump at a constant flow rate. This was to ensure CSWW would be provided sufficiently to the seed sludge-containing microbes.

### 2.3. Modified Synthetic Wastewater for Acclimatisation Process

The seed sludge used for UASB 2 was acclimatised with MSWW prepared in the Public Health Laboratory at Faculty of Engineering, Universiti Putra, Malaysia. The MSWW used in this study is a modified version of synthetic wastewater which has been described earlier in [28]. The COD, total nitrogen (TN) and phosphorus (P) concentration of the MSWW were 203,000 mg/L, 19,800 mg/L, and 4000 mg/L, respectively. It was developed to yield a C:N:P ratio of 50:5:1, as suggested in [29], to have a balanced nutrient composition and similar content with typical untreated domestic wastewater. During 30 days of the acclimatisation period, MSWW was prepared daily; the detailed composition is represented in Table 2.

### 2.4. Analytical Method

The effluent from both reactors was collected on a daily basis and measured for pH, the volume of biogas production, and COD concentration. Other parameters, which include IA/PA ratio, total ammonia nitrogen (TAN), FOG, and biogas composition for methane concentration, were analysed twice a week. Volatile fatty acid (VFA) was sent for analysis twice for each OLR during the reactor stable state. Mettler-Toledo AG (Schwarzenbach, Switzerland) was used to monitor the pH of the reactors. COD, TSSs, and VSSs were measured according to the Standard Method for the Examination of Water and Wastewater [30]. TAN was measured using a spectrophotometer (HACH DR 900, Agilent, Santa Clara, CA, USA), and Salicylate Powder Pillow Method 8155 was adopted. Total alkalinity (IA/PA) was determined using the titrimetric method with 0.02 N sulphuric acid (H_2_SO_4_). Biogas composition was analysed by a gas chromatograph (Agilent HP 6890 N) with a thermal conductivity detector (TCD) and an HP-PLOT-Q capillary column (30 m × 0.5 mm × 40 µm). VFAs were quantified in a Shimadzu GC 2010 gas chromatograph, equipped with a flame ionisation detector (FID) and a FameWax capillary column (30 m × 0.32 mm × 0.25 μm).

### 2.5. Energy Recovery

The analysis of energy recovery was carried out using experimental data obtained from one of the bioreactors which performed well. The optimum condition was determined based on the highest specific methane production (SMP) achieved and at a better quality of effluent produced. Despite the fact that there are a variety of biogas transformation options available, the usage of a biogas internal combustion engine coupled to an electrical generator was considered because it is a commercially available and simple technology application [31]. The calculation was first to convert the SMP in the unit of L CH_4_/gCOD_added_ into m^3^/kgCOD and kJ/kgCOD, as described in Khanal et al. [32] in Equations (1) and (2). Then, energy recovered in kWh is calculated using Equation (3).

(1) $$\mathrm{Energy}\;\mathrm{in}\;E\;\left(\frac{\mathrm{kJ}}{\mathrm{kgCOD}}\right)=\mathrm{SMP}\;\left(\frac{m^{3}}{\mathrm{kgCOD}}\right)\times 35846\;\left(\frac{\mathrm{kJ}}{m^{3}}\right)$$

(2) $$\mathrm{Energy}\;\mathrm{in}\;P\left(\frac{\mathrm{kWh}}{\mathrm{kgCOD}}\right)=E\;\left(\frac{\mathrm{kJ}}{\mathrm{kgCOD}}\right)\times 0.00028\;\left(\frac{\mathrm{kWh}}{\mathrm{kJ}}\right)$$

(3) $$\mathrm{Energy}\;\mathrm{in}\;P_{e}\;\left(\mathrm{kWh}\right)=P\left(\frac{\mathrm{kWh}}{\mathrm{kgCOD}}\right)\times \mathrm{COD}\;\mathrm{in}\;\left(\mathrm{kgCOD}\right)$$

where COD_in_ is the chemical oxygen demand of influent (kgCOD per day).

## 3. Results and Discussion

### 3.1. Acclimatisation of UASB 2 with MSWW

**Figure 3:**
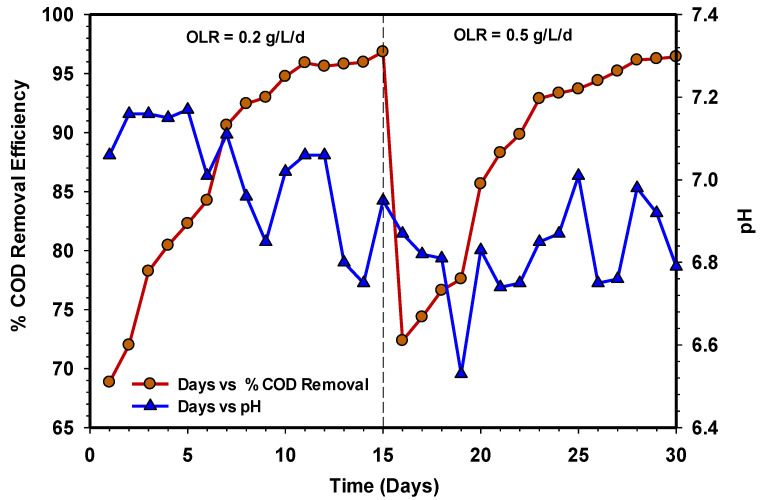
Acclimation period UASB 2 showing COD removal efficiency and pH profile.

Biomass acclimatisation of UASB 2 using MSWW was performed at a relatively low OLR (0.2–0.5 gCOD/L/d) for the adaptation process. The COD was evaluated at every stabilisation stage of each OLR. The acclimatisation stage lasted for one month before starting with feeding the real CSWW and compared with another reactor (UASB 1). Figure 3 depicts the COD removal efficiencies of UASB 2 during acclimatisation. The amount of COD removal in all the stages of the OLR was >90% stable state. Additionally, the pH profile of the system range between 6.5 to 7.2, which demonstrates a stable condition throughout the process.

### 3.2. COD Removal Efficiency

**Figure 4:**
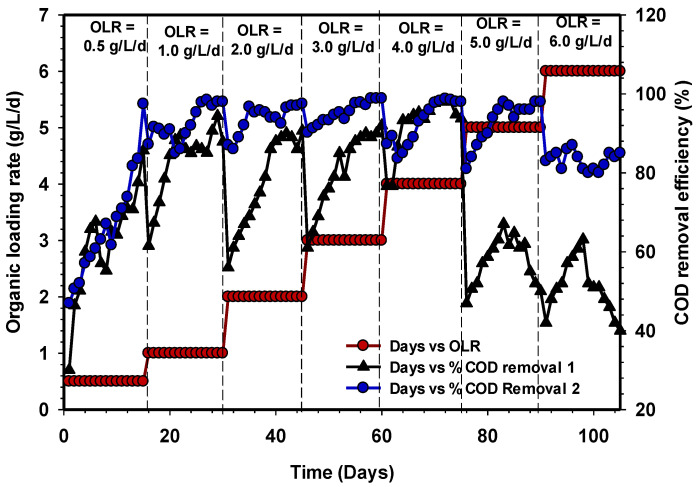
Variation in COD removal efficiency of UASB 1 and 2.

The COD removals were evaluated at various OLR to see the performance of the UASB reactors, as shown in Figure 4. The OLR varied from 0.5 to 6 gCOD/L/d during the whole period of the study. The COD removal efficiencies of the reactors at a steady state of OLR between 0.5 to 4 gCOD/L/d achieved more than 90% on average. However, there was a decline in COD removal efficiencies, along with the different phases of OLR before reaching a stable state [33].

Conversely, with increasing OLR to 5 and 6 gCOD/L/d, the reactor UASB 1 experienced a severe decline in COD removal to <70% at a stable state. The deficiency reported in UASB 1 could be related to the microbial population’s shock in coping with the shift in loading rate. On the other hand, UASB 2 maintained significant COD removal efficiency up to 85% at a stable state. The rigidity of UASB 2 is probably due to the microbial community that well adapt to the environment [34]. The study of Li et al. [35] further demonstrated that withstanding high OLR by the reactor is a function of microbial adaptability to the subject environment. Furthermore, the investigation of [36] had proven that once the operation is within the usual OLR boundaries, which range between 1.5 and 16.0 kgCOD/m^3^/d, a UASB reactor can sustain a stable process.

### 3.3. Biogas, Methane, and Specific Methane Productions (SMPs)

**Figure 5:**
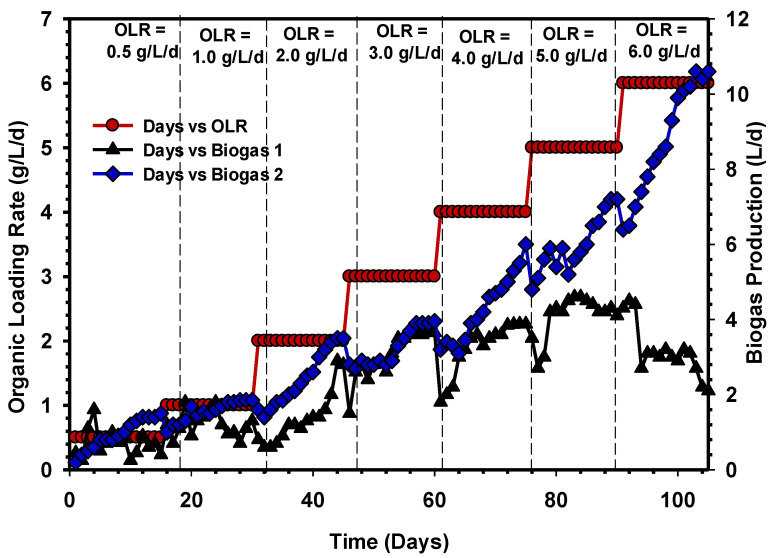
Daily biogas production over a period of operation of UASB 1 and 2.

The process performance of the UASB reactors in terms of biogas production is illustrated in Figure 5. The reactors continuously operated for 105 days with raw CSWW at various OLR, and as OLR increased, biogas output continued to rise. The systems experienced a slight decrease in the rate of biogas production once the OLR changed to another phase but gradually stabilised until the production rate remained the same for three or more consecutive days. This trend was maintained between 0.5 to 3 gCOD/L/d for both reactors, with the biogas production of 3.5 L/d in all the systems. However, the performance of UASB 1 drastically declined when the OLR increased to 4 gCOD/L/d (Figure 5), indicative of despair in the system. Likewise, the addition of OLR to 5 and 6 gCOD/L/d to both systems further deteriorated the performance of UASB 1 in terms of the daily biogas production, whereas UASB 2 proved to be more efficient and resistive to shock load, with a biogas production of 5.6, 7.2 and 10 L/d under the same condition of loading with UASB 1.

**Figure 6:**
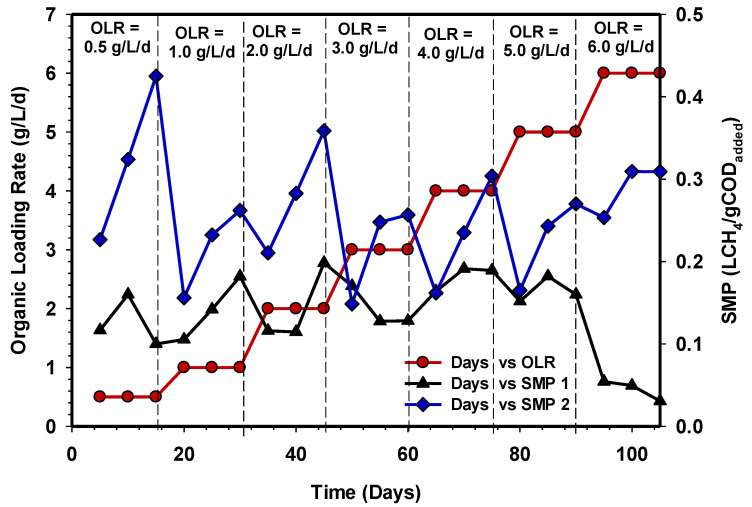
SMP over time in UASB 1 and 2.

The specific methane yield showed a similar trend, as demonstrated in Figure 6. It was observed that the SMPs of UASB 2 were considerably above 0.2 LCH_4_/gCOD_added_ from OLR 0.5 to 5 gCOD/L/d, while SMPs of UASB 1 were below 0.2 LCH_4_/gCOD_added_ at a steady state, under the same condition with UASB 2. However, increasing OLR to 6 gCOD/L/d showed a high rise in the SMPs produced by UASB 2 (0.31 LCH_4_/gCOD_added_), while a drastic reduction in SMPs to <0.05 LCH_4_/gCOD_added_ was observed in UASB 1. Studies on AD have revealed that changing feed techniques can improve biogas output and COD removal efficiency, which is the increasing or decreasing OLR. For instance, the investigation of Basitere et al. [37] showed that biogas and methane concentration improved with a gradual increment of OLR.

**Figure 7:**
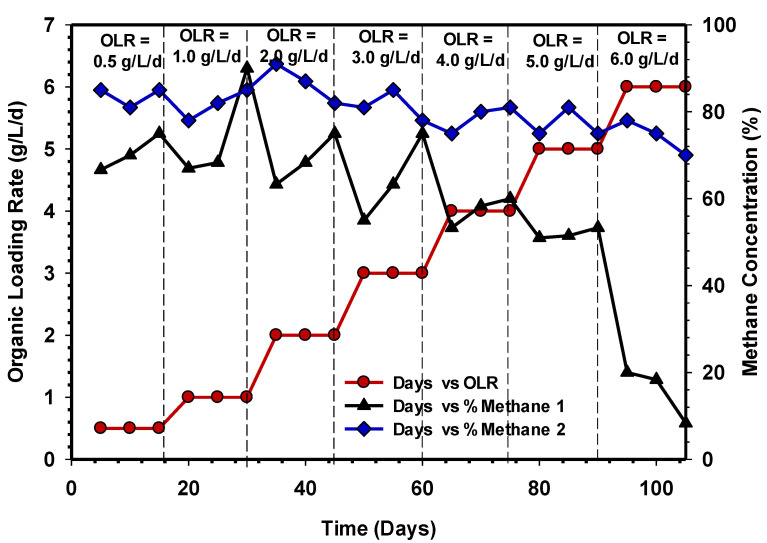
Methane concentration over time in UASB 1 and 2.

Figure 7 indicates the composition of methane gas concentration over a period of operation of UASB 1 and 2. It is clear that the dramatic drop in UASB 1 methane concentration was due to the shock the systems received as a result of the increases in OLR, which substantially harmed the methanogenic activity.

Furthermore, the reduction in methane concentration in UASB 1 indicates a sign of inhibition of the methanogens due to VFA accumulation, as well as a low pH. However, the UASB 2 system maintained a high concentration of methane above 70% throughout the different stages of OLR (Figure 7), and this depicts a stable state of the system with no sign of inhibition. Therefore, comparatively, the performance of UASB 1 containing non-acclimatised seed sludge revealed unstable methane gas production. The two experimental results revealed that UASB 2 is more efficient in biogas, methane, and SMP, compared with a UASB 1 reactor of the same configuration and different seed sludge. A similar result was reported by Torkian et al. [15] with a UASB reactor. Still, the results obtained from the previous study were lower (0.2 to 0.28 LCH_4_/gCOD_removed_) than UASB 2 in this study.

### 3.4. TAN Concentrations of the Reactors

**Figure 8:**
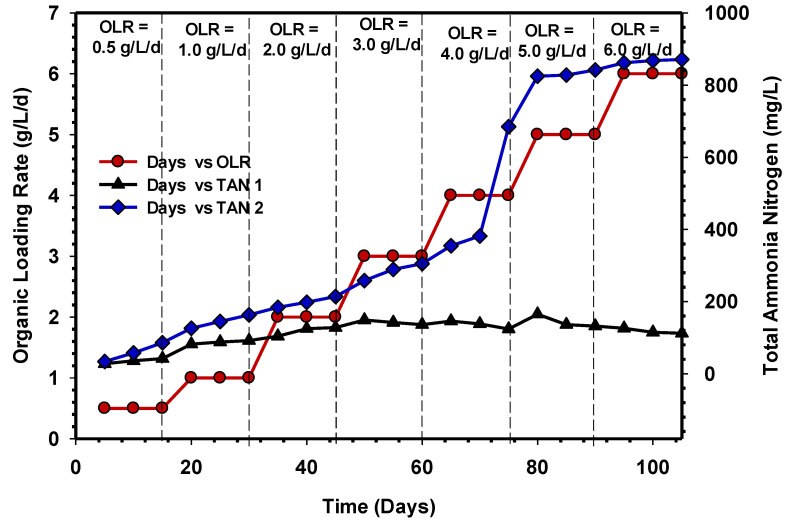
TAN concentration in UASB 1 and 2 over time.

The TAN concentration profile of UASB 1 and UASB 2 over a period of time at various OLRs are presented in Figure 8. Research has shown that during protein hydrolysis in AD, a high concentration of TAN is usually produced [38]. It can be inferred that the concentration of TANs for UASB 2 from OLR 0.5 to 3 gCOD/L/d were 86, 163, 214, and 305 mg/L, respectively. Meanwhile, the concentration of TANs in UASB 1 were found as 42, 92, 128 and 136 mg/L for OLR 0.5 gCOD/L/d to 3 gCOD/L/d, respectively.

Nonetheless, a significant difference was seen when the OLR was increased to 4, 5, and 6 gCOD/L/d to reactors, in which the values of TAN in UASB 2 increased from 685 mg/L at 4 gCOD/L/d to 813 mg/L at 6 gCOD/L/d, while it decreased from 135 mg/L at 4 gCOD/L/d to 112 mg/L at 6 gCOD/L/d in the UASB 1 reactor. Consequently, the differences that occurred as a result of increasing OLR in both reactors depict that protein hydrolysis was not occurring in UASB 1, and therefore, assimilation of COD by microbial population did not show progress, indicating that microorganisms (acetogens and methanogens) were not growing. Furthermore, the rapid breakdown of organic matter by the vast population of microbial biomass could be explained by the high production of TAN in the UASB 2 reactor. Comparatively, at low OLR, the performances of the reactors at steady states were quite stable. Nevertheless, after increasing the concentration of CSWW, UASB 2 demonstrated higher degradation of the substrate, indicating that the reactor was more efficient than UASB 1 at converting organic matter to methane at greater OLR. On the other hand, the TAN concentration in UASB 2 reactor’s effluent remained stable and did not hinder or create any substantial changes in the reactor’s functioning at higher OLR.

### 3.5. IA/PA Ratio and pH Profile Variations of the UASB Reactors

**Figure 9:**
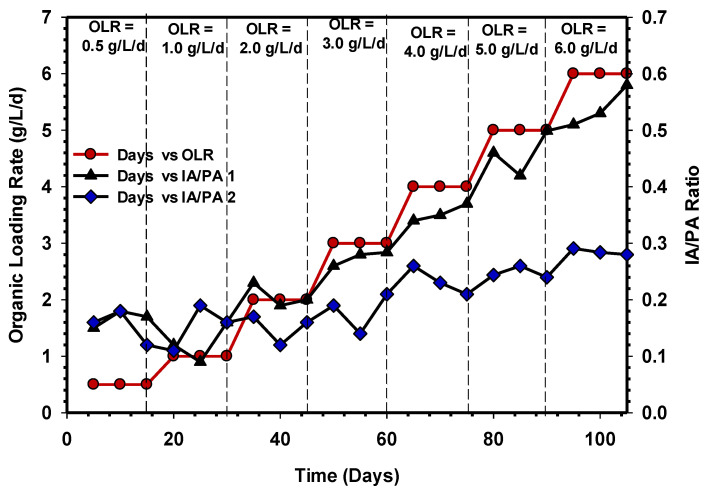
Variation of IA/PA ratio of UASB 1 and 2.

During AD, the IA/PA value determines the system’s ability to survive variations in pH caused by the release of organic acids [39]. Figure 9 represents the IA/PA ratio of UASB 1 and 2 during the treatment of CSWW over time. The wastewater is characterised by high protein and lipid content and thus very challenging when subjected to AD. According to Tangkathitipong et al. [40], during AD, the IA/PA ratio of the reactor has to be maintained in the range of 0.1 to 0.3; this is because when the ratio of IA/PA exceeds 0.4, the system is likely to undergo unstable condition [8].

**Figure 10:**
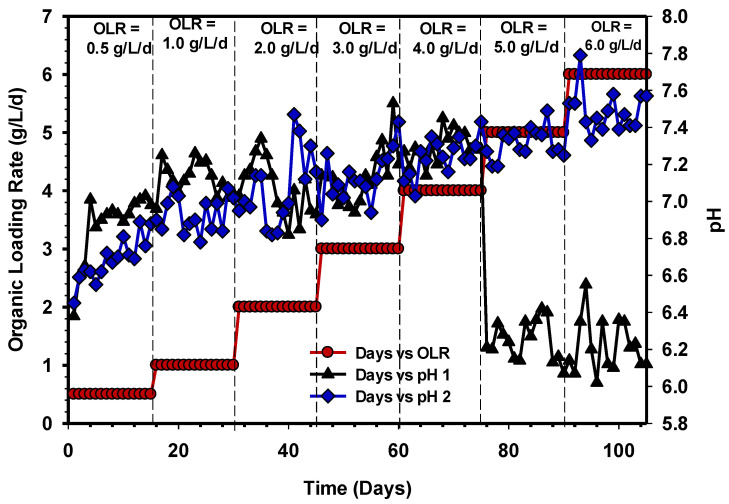
pH profile of UASB 1 and 2 reactors.

In this research, it was observed that the IA/PA ratio from OLR 0.5 to 3 gCOD/L/d was <0.3, in both UASB 1 and 2 (Figure 9). The numbers indicate a stable working environment with enough alkalinity, which is substantially below the inhibitory level. Subsequently, an increase in the OLR 4 to 6 gCOD/L/d in UASB 1 resulted in an increase in IA/PA ratio to above 0.3, and this signifies the abnormal function of the system. Moreover, the inhibition that occurred in UASB 1 could be due to the long-chain fatty acids (LCFAs) accumulated in solid biomass with a low pH, as shown in Figure 10. The investigation of [41] also supports the findings of this study. The IA/PA ratio values of the UASB 2 reactor were maintained below 0.3 at OLR 4, 5, and 6 gCOD/L/d (Figure 9), indicating a stable state of AD. Figure 10 demonstrated the pH profile of UASB 1 and 2 at different OLRs.

During AD, microorganisms have a working range of pH between 6.5 and 7.5 [42]. According to Morales-Polo et al. [43], fermentative bacteria strive around pH 4 to 8.5, while methanogenic bacteria performed well around 6.5 and 7. The pH of both reactors was within a stable state at OLR 0.5 to 4 gCOD/L/d. However, when the influent concentration increased, the pH profiles at 5 to 6 gCOD/L/d OLR of UASB 1 drastically declined (Figure 10), while UASB 2 maintained a stable condition. Furthermore, the pH values in UASB 2 were stable with the increased levels of OLR. As a result, the effluent of the UASB 2 reactor has a high buffering capacity and does not require pH correction.

### 3.6. Fats, Oil, and Grease (FOG) Concentration in the UASB Reactors

**Figure 11:**
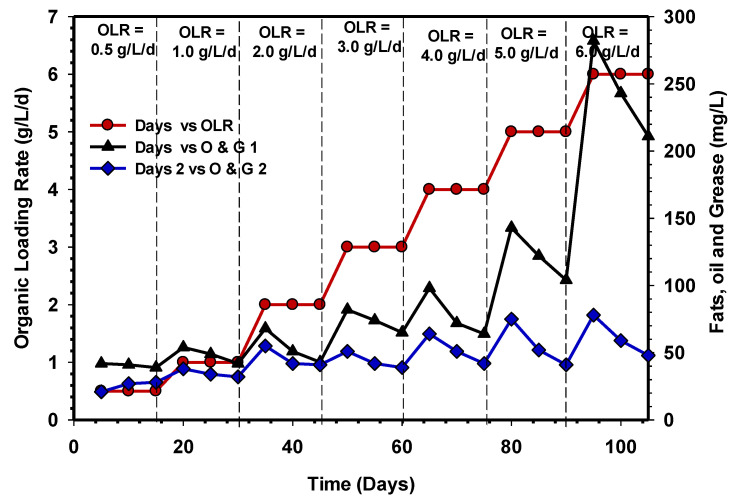
Comparison of FOG removal efficiencies of UASB 1 and 2.

Figure 11 demonstrates the FOG pattern removal from the two UASB reactors. CSWW is classified as a high-strength waste stream due to its refractory character. On the other hand, studies have shown that at higher OLR, FOG may cause significant digester foaming in the reactor due to reactor inhibition [44]. Moreover, according to Jeganathan et al. [45], the presence of high FOG in an anaerobic reactor could result in the accumulation of scum that affects degradation, especially in UASB reactors.

It was observed that an increase in OLR from 0.5 to 4 gCOD/L/d did not negatively affect the performance of UASB 2, nor did the subsequent loading rates (5–6 gCOD/L/d) (Figure 11). It can be seen that FOG in UASB 1 increased with increasing OLR from 4, 5, and 6 gCOD/L/d (Figure 11). The trend of FOG development in UASB 1 suggested that no assimilation of organic matter occurred, which could be attributed to LCFAs inhibiting propionate breakdown and a high propionate concentration causing inhibiting in hydrolysis [46]. However, according to [47,48], reactor failure is probably caused by the inhibition of methanogens and acetogens, whereas the research of Jensen et al. [49] suggest that when LCFAs exit the environment and accumulate in solid biomass within 24 h, they are adsorbed into the membrane/cell wall of bacteria, affecting the microbial cell’s ability to transport or protect itself.

### 3.7. VFA Formation in the UASB Reactors

**Figure 12:**
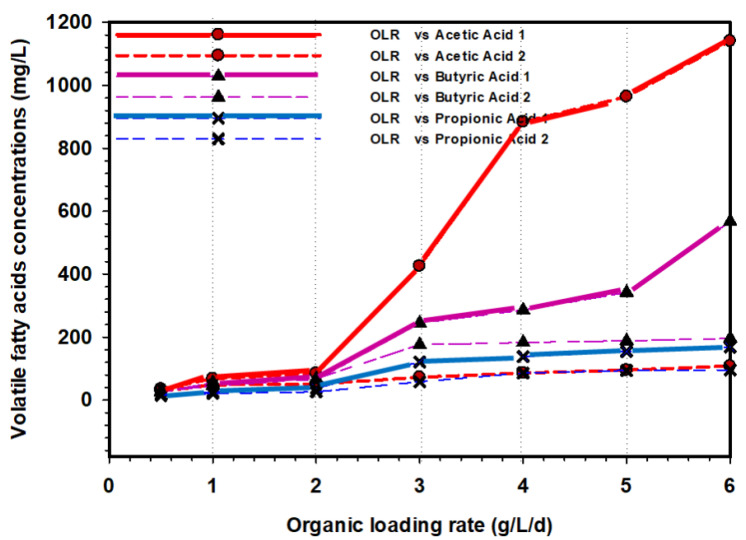
Comparison of VFA formation profiles in UASB 1 and 2.

Figure 12 represents the VFA concentrations in mg/L over a period of time during the performance comparison of UASB 1 and 2. The samples were analysed once in every stage of OLR (0.5–6 gCOD/L/d). A relatively low VFA concentration was recorded from OLR 0.5 to 2 gCOD/L/d, as shown in Figure 12. These demonstrate that methanogenic activities proceed effectively. When the OLR was increased to 3, 4, 5, and 6 gCOD/L/d in the UASB 1 reactor, a rapid increase in acetic, propionic, and butyric acids was observed. By contrast, in the UASB 2 reactor under the same condition of loading, no significant changes in acetic, propionic, and butyric acids were observed. This indicates that the experiment was extremely stable during these times. In addition, the UASB 2 reactor’s performance demonstrated that the microbial population had successfully acclimatised to the system.

The rise in the acetic acid in UASB 1 when the OLR was further increased to 3, 4, 5, and 6 gCOD/L/d could be due to the drop in pH and subsequent accumulation of VFA, which led to inhibition. Furthermore, the investigation of Ugurlu and Forster [50] revealed that an increase in feed concentration could cause the reactors under pressure that usually showed a drop in pH and VFAs accumulation [51]. It was observed that after increasing OLR in UASB 1, the effluent had become completely acidified, which suggests a high presence of acidogens and acetogens without substrate assimilation. Similarly, studies have revealed that the suppression of methanogens and acetogens is the primary cause of reactor failure [52,53]. Likewise, the build-up and absorption of LCFAs inhibit the microbial cell transport system when LCFAs enter the membrane or cell wall of bacteria [54].

### 3.8. Summary of Bioreactor Performances and Energy Recovery from Laboratory-Scale and Commercial-Scale Anaerobic Reactor

In this study, the energy recovery for LS and CS were evaluated to determine the potential power yield, as illustrated in Table 3. The energy recovery was calculated based on the methane yield or SMP achieved in LS at optimum conditions. OLR 5 g/L/d for UASB 2 was selected as the optimum phase due to the high performance achieved and better quality of effluent produced. The SMP recorded at this phase was 0.27 LCH_4_/gCOD_added_, as shown in Table 4. The projection of energy recovery for CS was calculated based on the SMP obtained in LS. As presented in Table 1 in the earlier subsection, the COD concentration of CSWW was 14.35 g/L. CS reactor is to be operated at a wet market. The average number of slaughtered chickens is 1000 per day at a wet market located in Seri Kembangan, Selangor, Malaysia. Taking CSWW produced for one chicken into account, the value is 20.49 L [55]. Therefore, it is estimated that the CSWW production for that one day is 20,490 L.

The energy produced from LS and CS is 0.0562 kWh and 790.49 kWh, respectively. Wresta et al. [26] reported that only 2 kWh from a total of 6 kWh can be converted into electricity due to energy losses during the treatment and conversion process. This is equivalent to only 33% of the energy that can be recovered and utilised as electricity. Therefore, the power yield for LS and CS are 0.0185 kWh and 260.86 kWh, respectively, considering the SMP value is constant with the percentage of methane is within the range of 70% to 75%. The energy generated (energy yield) can be utilised for the internal operation of the wet market.

Furthermore, energy generated can also be used for slaughtering activities, thus reducing the grid energy. For instance, based on the technical specifications of commercial products, the power consumption for certain standard slaughterhouse equipment is shown in Table 5.

**Figure 13:**
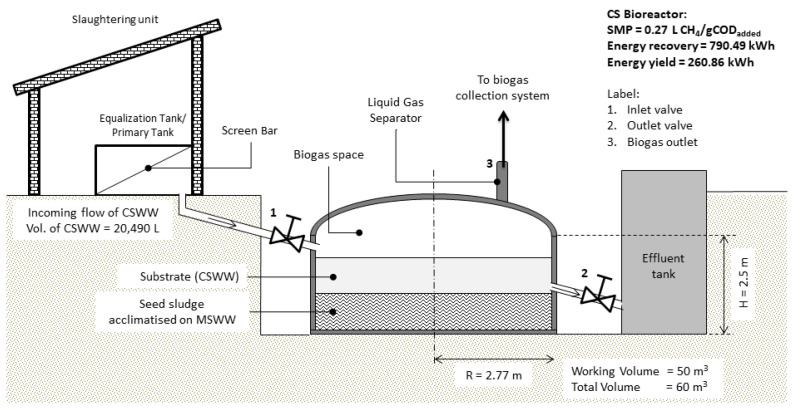
Process flow diagram of on-site CS CSWW treatment.

Assuming the operation at the wet market slaughterhouse is about 8 h per day, the total power consumed is at least 44 kWh, excluding the use of a hydraulic pump, since the CSWW will gravitationally flow into the CS reactor. The CS reactor is designed in a cylindrical shape with 2.5 m of height and 2.77 m in radius, yielding a total volume of 60 m^3^, equivalent to 60,000 L to sustain the feeding of 20,490 L of CSWW produced per day. Considering 50% of working volume (W_v_) is seeded with sludge and 50% with CSWW, the proposed W_v_ of CS is 50,000 L. Headspace is allowed in the CS reactor, thus making the total volume equal to 60,000 L, equivalent to 60 m^3^. The CS reactor comprised a biogas digester, biogas dome, and gas–liquid separator for biogas collection. In addition, the equalisation tank for the CSWW collection is equipped with screen bars to sieve any suspended particles with feathers and carcasses to avoid system clogging. The proposed hydraulic retention time (HRT) was 9.5 h to allow a sufficient period for the substrate to degrade with low V_up_. CSWW flows into the CS reactor from the top of the digester to break any scum formation and allows the substrate to properly distribute into the sludge blanket, thus minimising the maintenance cost [57]. Furthermore, the CS reactor is designed to be operated at ambient temperature between 31 and 35 °C since Malaysia has a tropical climate throughout the year. Therefore, no external heating system is required. Figure 13 shows the process flow diagram of CS CSWW treatment.

## 4. Conclusions

The performance of UASB 1 and 2 reactors in terms of biogas production and other physicochemical parameters were studied. The UASB 2 reactor acclimatised with MSWW showed excellent efficiency at all the OLRs applied throughout the study period. The efficiency of the system was attributed to the presence of high microbial growth after acclimatisation with MSWW. Thus, the system was able to withstand high OLR without a sign of inhibition. Conversely, the performance of UASB 1 declined drastically in all study parameters when the OLR was increased to 3, 4, 5, and 6 gCOD/L/d. The methane content in UASB 2 was stable (>80%) when the OLR increased. The optimum phase for UASB 2 was recorded at OLR 5 gCOD/L/d, in which the SMP was reported at 0.27 LCH_4_/gCOD_added_ and COD concentration of the effluent at 0.16 g/L, which meet the standard (B) for water quality discharge parameters set by the Department of Environment (DOE) Malaysia. Energy recovery projected in the CS bioreactor indicates the system is capable to sustain the power needed for wet market operation. The energy yield recorded in CS was 260.86 kWh, which is higher than the energy consumed in the slaughtering unit, 44 kWh. Considering the actual daily volume of CSWW generated, detailed assessment of process performance study, and existing design of LS system, the CS anaerobic digester was proposed at a capacity of 60 m^3^.

## Figures and Tables

**Table 1:** The initial composition of CSWW.

Parameters	Unit	Average Value
pH	-	7.08 ± 0.50
Temperature	°C	26.7 ± 0.5
COD	mg/L	14,350 ± 300
TSSs	mg/L	2335 ± 100
VSSs	mg/L	1524 ± 150
FOG	mg/L	6820 ± 100
TN	mg/L	3033 ± 200
Colour	Pt–Co	12,300 ± 100
Turbidity	FAU	8500 ± 50

COD: chemical oxygen demand; TSSs: total suspended solids; VSSs: volatile suspended solids; FOG: fats, oil, and grease; TN: total nitrogen; Pt–Co: platinum–cobalt scale; FAU: formazin attenuation units.

**Table 2:** MSWW composition.

Materials	Quantity	Unit
Yeast (granular form)	23	g
Urea	2	g
Ammonium phosphate (NH_4_)2HPO_4_	3.4	g
Condensed Milk	140	mL
Chicken’s pure blood	5.75	mL
Tap water	To make up to 1 L	

**Table 3:** Energy recovery from the treatment of CSWW in LS and CS.

Parameter	Unit	LS	CS
OLR	gCOD/L/day	5.0	5.0 ^b^
SMP	LCH_4_/gCOD	0.27	0.27 ^b^
Energy in kJ/kgCOD ^a^	kJ/kgCOD	9678.42	9678.42
Energy in kWh/kgCOD ^a^	kWh/kgCOD	2.69	2.69
Energy Recovery in kWh	kWh	0.0562	790.49
Energy yield in kWh	kWh	0.0185	260.86

^a^: Assuming calorific value of CH_4_ at standard temperature pressure = 35,846 MJ/m^3^ and 1 kJ = 0.00028 kWh/kJ [32]; ^b^: assuming optimum OLR and SMP in CS equal to LS.

**Table 4:** Summary of UASB 1 and UASB 2 performance comparison.

OLR (gCOD/L/day)	COD in (g/L)	COD out (g/L)		COD Removal (%)		Biogas Production ( L)		Methane Content (%)		SMP (LCH_4_/gCOD_added_)		pH		IA/PA Ratio		TAN (mg/L)		TSSs (mg/L)		FOG (mg/L)		Colour (Pt-Co)	
1	2	1	2	1	2	1	2	1	2	1	2	1	2	1	2	1	2	1	2	1	2	1	2
0.5	0.86	0.12	0.14	85.7	83.6	0.73	1.40	75	85	0.10	0.43	7.01	6.84	0.170	0.120	42	86	10	7	39	28	86	95
1.0	1.71	0.10	0.03	94.4	98.0	1.03	1.85	84	85	0.18	0.26	7.05	6.98	0.130	0.160	92	163	25	11	42	32	81	90
2.0	3.43	0.14	0.08	96.0	97.5	2.17	3.47	77	82	0.20	0.36	6.97	7.19	0.200	0.160	128	214	36	21	50	41	71	82
3.0	4.80	0.06	0.06	98.9	98.7	3.63	3.92	78	85	0.17	0.25	7.29	7.25	0.281	0.210	136	305	51	32	65	40	62	89
4.0	6.40	0.13	0.13	98.0	98.0	3.88	5.60	59	81	0.19	0.30	7.28	7.32	0.370	0.210	135	685	65	26	68	50	55	84
5.0	8.00	2.53	0.16	69.1	98.0	4.20	7.13	53	75	0.16	0.27	6.12	7.27	0.462	0.240	136	805	76	32	121	51	56	75
6.0	9.60	4.48	1.44	53.3	85.0	2.33	10.53	20	70	0.03	0.31	6.16	7.52	0.540	0.280	112	813	403	98	245	52	42	78

OLR: organic loading rate; COD: chemical oxygen demand; SMP: specific methane production; IA/PA: alkalinity ratio; TAN: total ammonia nitrogen; TSSs: total suspended solids; FOG: fats, oil, and grease; HRT: hydraulics retention time; 1: UASB 1; 2: UASB.

**Table 5:** Equipment used during slaughtering activity and power consumption.

Equipment	Power Required (kW)	Power Consumption in 1-Day Operation 8 h (kWh)
Boots cleaning ^c^	2	16
Lights and office equipment ^c^	2	16
Feather removal machine ^d^	1.5	12
Total power consumption		44

^c^: [5]; ^d^: [56]^.^

## Data Availability

Not applicable.
